# Methylene blue for clinical anaphylaxis treatment: a case report

**DOI:** 10.1590/S1516-31802007000100012

**Published:** 2007-01-04

**Authors:** Janine Moreira Rodrigues, Antonio Pazin, Alfredo José Rodrigues, Walter Vilella de Andrade Vicente, Paulo Roberto Barbosa Evora

**Keywords:** Anaphylaxis, Nitric oxide, Methylene blue, Guanylate cyclase, Cyclic GMP, Anafilaxia, Óxido nítrico, Azul de metileno, Guanilato ciclase, GMP cíclico

## Abstract

**CONTEXT AND OBJECTIVE:**

Nitric oxide has a pathophysiological role in modulating systemic changes associated with anaphylaxis. Nitric oxide synthase inhibitors may exacerbate bronchospasm in anaphylaxis and worsen clinical conditions, with limited roles in anaphylactic shock treatment. The aim here was to report an anaphylaxis case (not anaphylactic shock), reversed by methylene blue (MB), a guanylyl cyclase inhibitor.

**CASE REPORT:**

A 23-year-old female suddenly presented urticaria and pruritus, initially on her face and arms, then over her whole body. Oral antihistamine was administered initially, but without improvement in symptoms and signs until intravenous methylprednisolone 500 mg. Recurrence occurred after two hours, plus vomiting. Associated upper respiratory distress, pulmonary sibilance, laryngeal stridor and facial angioedema (including erythema and lip edema) marked the evolution. At sites with severe pruritus, petechial lesions were observed. The clinical situation worsened, with dyspnea, tachypnea, peroral cyanosis, laryngeal edema with severe expiratory dyspnea and deepening unconsciousness. Conventional treatment was ineffective. Intubation and ventilatory support were then considered, because of severe hypoventilation. But, before doing that, based on our previous experience, 1.5 mg/kg (120 mg) bolus of 4% MB was infused, followed by one hour of continuous infusion of another 120 mg diluted in dextrose 5% in water. Following the initial intravenous MB dose, the clinical situation reversed completely in less than 20 minutes, thereby avoiding tracheal intubation.

**CONCLUSION:**

Although the nitric oxide hypothesis for MB effectiveness discussed here remains unproven, our intention was to share our accumulated cohort experience, which strongly suggests MB is a lifesaving treatment for anaphylactic shock and/or anaphylaxis and other vasoplegic conditions.

## RESUMO

**CONTEXTO E OBJETIVO:**

O óxido nítrico (NO) tem papel fisiopatológico na modulação das alterações sistêmicas associadas à anafilaxia. Inibidores da sintase do NO podem exacerbar a broncoconstrição na anafilaxia e piorar as condições clínicas e têm um papel limitado no tratamento do choque anafilático. O objetivo deste relato foi o de apresentar um caso de anafilaxia (não-choque anafilático) revertido pela inibição da guanilato ciclase pelo azul de metileno.

**RELATO DE CASO:**

Mulher de 23 anos apresentou repentina urticária e prurido, inicialmente localizados na face e nos braços, que se estenderam para quase todo o corpo. Utilizou-se, inicialmente, anti-histamínico, mas pequena melhora só foi observada após o uso endovenoso de 500 mg metilprednisolona. Após duas horas, os sintomas voltaram acompanhados de vômitos. Observou-se dificuldade respiratória alta, sibilos respiratórios, estridores laríngeos, angioedema facial, incluindo eritema, edema de lábios e, nos locais de intenso prurido, observaram-se petéquias. O quadro agravou-se com dispnéia, taquipnéia, parestesia peroral, edema laríngeo, grave dispnéia expiratória com crescente perda da consciência. O tratamento convencional não foi efetivo. Considerou-se intubação pela grave hipoventilação. Com base em nossa prévia experiência, um bolo endovenoso de 1,5 mg/kg (120 mg) de azul de metileno a 4% diluído em soro glicosado 5%, seguido de outros 120 mg infundidos durante uma hora. Após a dose endovenosa inicial do azul de metileno, a situação clínica reverteu-se completamente, evitandose a intubação traqueal.

**CONCLUSÃO:**

Embora a hipótese da eficácia da relação entre o óxido nítrico e o azul de metileno ainda esteja por ser provada, nossa intenção foi de compartilhar nossa experiência acumulada, que sugere fortemente que o azul de metileno seja um tratamento salvador de vidas para o choque anafilático e/ou anafilaxia e outras condições vasoplégicas.

## INTRODUCTION

Nitric oxide (NO) seems to play an important pathophysiological role in modulating the systemic changes associated with anaphylaxis. Nitric oxide synthase (NOS) inhibitors attenuate hypotension and blood concentration and decrease venous returns under such conditions, although they do not improve cardiac depression. On the other hand, NO functionally antagonizes the effects of the vasoconstrictors released during anaphylaxis *in vitro*, and there is experimental evidence that NO production may reduce some pathophysiological changes associated with anaphylaxis, except for vasodilatation.^1^

The use of NOS inhibitors in experimental therapy for anaphylactic shock is questionable. NOS inhibitors may improve blood pressure, but there is a massive reduction in cardiac output at the same time. Moreover, NO produced by the bronchial epithelium may play an important role in counteracting anaphylactic bronchospasm. Thus, NOS inhibitors may exacerbate bronchospasm in anaphylaxis and worsen the clinical condition. Taken together, it appears that NOS inhibitors may have a limited role in therapy for anaphylactic shock, in comparison with endotoxic, septic or hemorrhagic shock, because of differences in pathophysiological mechanisms, the nature of the NOS isoforms involved, and differences in clinical presentation. By stimulating soluble guanylyl cyclase, nitric oxide increases cyclic guanosine 3’,5’-monophosphate (GMP) production and leads to smooth muscle relaxation. On the other hand, methylene blue does not interfere with NO release but acts to block its action on vascular smooth muscles.^2,3^

The aim of this paper was to report a case of anaphylaxis (not anaphylactic shock) that was reversed by methylene blue, a guanylyl cyclase inhibitor.

## CASE REPORT

A 23-year-old female medical student who was working as a urological video laparoscopy assistant suddenly presented urticaria and pruritus, initially on her face and arms, and then extending quickly to her whole body. No allergen could be identified and there was no previous personal history of allergy, but retrospective hypotheses included allergy due to surgical glove talc or inhalation of the glutaraldehyde that is used as a conservative solution for biological material.

The first medication given was oral antihistamine, but a mild improvement in symptoms and signs was only detected after administering intravenous methylpredniso-lone 500 mg. However, two hours after the first episode and medications, the symptoms and signs returned with the addition of vomiting. Associated upper respiratory distress, pulmonary sibilance, laryngeal stridor and significant facial angioedema (including erythema and lip edema) marked the evolution. At sites with severe pruritus, petechial lesions were observed. The clinical situation worsened, with dyspnea, tachypnea, peroral cyanosis, laryngeal edema with severe expiratory dyspnea and deepening unconsciousness. The patient was not in a state of circulatory shock, but the urticarial lesions, angioedema and upper respiratory tract distress continued to worsen.

Conventional treatment (300 mcg adrenaline injections and 1.0 g hydrocortisone), associated with other drugs like antihistamines, midazolam (20 mg), morphine (5 mg) and aerosols of beta-2 adrenergic agonists failed to reverse the imminent cardiocirculatory collapse. General sedation and curare for lung intubation and ventilatory support were considered at that moment, in view of the severe hypoventilation (PO_2_ = 168 mmHg and PCO_2_ = 217 mmHg). But before undertaking this extreme solution, and based in our previous clinical and laboratory experience, a 1.5 mg/kg (120 mg) bolus of 4% methylene blue was administered, followed by one hour of continuous infusion of another 120 mg diluted in 5% dextrose 5% in water. An initial bolus dose was selected because of the severity of the anaphylaxis. Following the initial dose of intravenous methylene blue, the clinical situation reversed completely (angioedema, urticaria, vasodilatation and upper respiratory dyspnea) in less than 20 minutes, thereby avoiding tracheal intubation. Unfortunately, no photograph was taken before the use of methylene blue, when the angioedema was very severe. Nonetheless, it was still present in the eyelids 20 to 30 minutes after treatment. Around 60 minutes later, the patient did not presented any anaphylaxis symptom apart from slight eyelid edema. No new drugs were adminis tered, and no drugs used previously were repeated. The patient was discharged home two hours later.

## DISCUSSION

We previously reported our initial cumulative clinical experience with a cohort of nine patients who developed anaphylactic shock and/or anaphylaxis and were treated with an intravenous bolus of methylene blue (1.5 to 2.0 mg/kg). As anaphylaxis and anaphylactic shock are human emergencies, and as we do not have any evidence to propose methylene blue as a first-choice drug, it is very difficult or impossible to design a randomized study that would be in accordance with ethical principles.

Among the total of our nine patients mentioned in the preceding paragraph, two did not present circulatory collapse and only presented the major signs of anaphylaxis, as in the present case report. It is important to differentiate between the two situations (anaphylaxis and anaphylactic shock) that were reversed by methylene blue. This emphasis is based on the possibility of methylene blue use in cases of anaphylactic reactions without cardiovascular collapse. The present case report is the third case of anaphylaxis without shock in a series of nine reported cases.^2,3^

**Figure 1:**
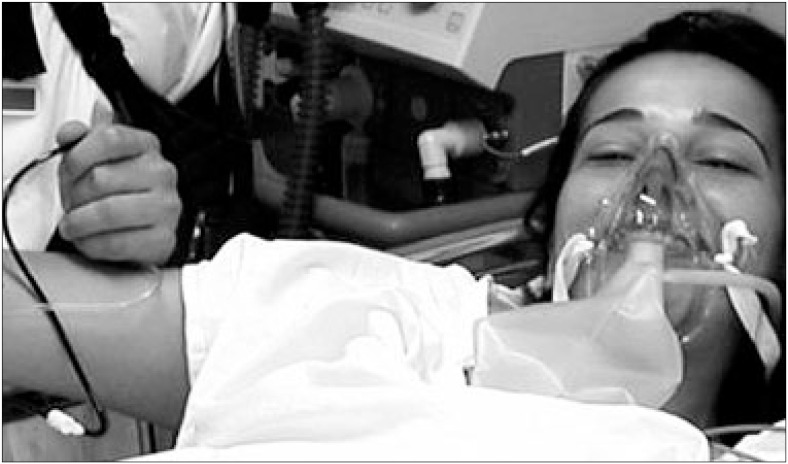
Photograph of patient 20 to 30 minutes after administering the intravenous bolus of methylene blue (1.5-2 mg/kg); facial angioedema can still be seen. The patient kindly gave permission for this picture to be published

Side effects in the form of nodal rhythm and chest pain have been observed in one patient each. The nodal rhythm that occurred during methylene blue infusion reverted spontaneously to normal rhythm within a minute. Another hypertensive patient, who had anaphylactic shock during computed tomography scan, complained of chest pain during the methylene blue infusion but, as no electrocardiographic changes ensued, coronary vasodilators were not utilized. We stress that the physiopathology of the electrocardiographic changes (nodal rhythm) and the chest pain episode could be attributed either to the methylene blue infusions or to the radiocontrast agent used.^2,3^

## CONCLUSIONS

Further research is warranted to evaluate the role of methylene blue. On the basis of the abovementioned clinical experience, we have designed a research line for studying anaphylactic shock treatment experimentally, which is currently one of our goals. Considering the lack of knowledge, the experimental protocols that we have started with have produced good responses.^4,5^

These observations do not allow it to be assumed that methylene blue would be the first-choice drug for anaphylactic shock treatment. Moreover, it has to be emphasized that adrenaline remains the drug of choice. Nonetheless, we can speculate about synergism between these two drugs, since this association acts to stimulate the cyclic adenosine 5’- monophosphate (AMP) system and block the cyclic GMP system, thereby counterbalancing vasoplegia. Although the nitric oxide hypothesis for the effectiveness of methylene blue discussed here still remains unproven, our intention has been to share our accumulated cohort experience, which strongly suggests that methylene blue is a lifesaving treatment for anaphylactic shock and/or anaphylaxis and other vasoplegic conditions.
